# Understanding the complexity of glycaemic health: systematic bio-psychosocial modelling of fasting glucose in middle-age adults; a DynaHEALTH study

**DOI:** 10.1038/s41366-018-0175-1

**Published:** 2018-08-17

**Authors:** Estelle Lowry, Nina Rautio, Ville Karhunen, Jouko Miettunen, Leena Ala-Mursula, Juha Auvinen, Sirkka Keinänen-Kiukaanniemi, Katri Puukka, Inga Prokopenko, Karl-Heinz Herzig, Alexandra Lewin, Sylvain Sebert, Marjo-Riitta Järvelin

**Affiliations:** 1 Center for Life Course Health Research, P.O. Box 5000, 90014 University of Oulu, Oulu, Finland; 2 Biocenter Oulu, University of Oulu, P.O. Box 5000, 90014 Oulu, Finland; 3 Unit of Primary Health Care, Oulu University Hospital, P.O. Box 20 90029 OYS, Oulu, Finland; 4 Department of Epidemiology and Biostatistics, Imperial College London, St. Mary’s Campus, W2 1PG London, United Kingdom; 5 Medical Research Center Oulu, Oulu University Hospital and University of Oulu, P.O. Box 5000, 90014 University of Oulu, Oulu, Finland; 6 NordLab Oulu, Medical Research Center Oulu, Oulu University Hospital and Department of Clinical Chemistry, University of Oulu, Oulu, Finland; 7 Section of Genomics of Common Disease, Department of Medicine, Imperial College London, Hammersmith Hospital Campus, Burlington Danes Building, Du Cane Road, London, W12 0NN UK; 8 Department of Gastroenterology and Metabolism, Poznan University of Medical Sciences, Poznan, Poland; 9 Brunel University London, Kingston Lane, Uxbridge, Middlesex UB8 3PH United Kingdom; 10 MRC-PHE Centre for Environment and Health, Imperial College London, Praed Street Wing, St Mary’s Campus, Paddington, W2 IPG London United Kingdom

**Keywords:** Risk factors, Metabolic syndrome, Type 2 diabetes

## Abstract

**Background:**

The prevention of the risk of type 2 diabetes (T2D) is complicated by multidimensional interplays between biological and psychosocial factors acting at the individual level. To address the challenge we took a systematic approach, to explore the bio-psychosocial predictors of blood glucose in mid-age.

**Methods:**

Based on the 31-year and 46-year follow-ups (5,078 participants, 43% male) of Northern Finland Birth Cohort 1966, we used a systematic strategy to select bio-psychosocial variables at 31 years to enable a data-driven approach. As selection criteria, the variable must be (i) a component of the metabolic syndrome or an indicator of psychosocial health using WHO guidelines, (ii) easily obtainable in general health check-ups and (iii) associated with fasting blood glucose at 46 years (*P* < 0.10). Exploratory and confirmatory factor analysis were used to derive latent factors, and stepwise linear regression allowed exploration of relationships between factors and fasting glucose.

**Results:**

Of all 26 variables originally considered, 19 met the selection criteria and were included in an exploratory factor analysis. Two variables were further excluded due to low loading (<0.3). We derived four latent factors, which we named as socioeconomic, metabolic, psychosocial and blood pressure status. The combination of metabolic and psychosocial factors, adjusted for sex, provided best prediction of fasting glucose at 46 years (explaining 10.7% of variation in glucose; *P* < 0.001). Regarding different bio-psychosocial pathways and relationships, the importance of psychosocial factors in addition to established metabolic risk factors was highlighted.

**Conclusions:**

The present study supports evidence for the bio-psychosocial nature of adult glycemic health and exemplifies an evidence-based approach to model the bio-psychosocial relationships. The factorial model may help further research and public health practice in focusing also on psychosocial aspects in maintaining normoglycaemia in the prevention of cardio-metabolic diseases.

## Introduction

In 2015, diabetes caused an estimated 1.6 million deaths and ‘higher-than-optimal’ blood glucose was responsible for a further 2.2 million, through both direct clinical progression and as a risk factor for cardiovascular and kidney diseases [1]. Pivotally, age-standardised mean fasting plasma glucose has increased globally by 0.07 mmol/L per decade or more [2]. The global deterioration of glycaemic health highlights a need to address this problem before it reaches the clinical stages of disease.

Fasting plasma glucose generally follows a relatively stable linear trajectory and has been observed only to steeply increase up to 3 years before onset of diabetes [3]. Thus, preserving normal fasting plasma glucose levels may be key to maintaining good metabolic health and substantially delay diabetes onset.

The pathways leading to deterioration of glycaemic health are more complex than a linear cause-effect model may suggest. Maintaining normoglycaemia may rely on the interplay of diverse causal factors, including those at a biological, socioeconomic and psychosocial level. This follows the theory of the bio-psychosocial model for biomedicine crystallised by Engel four decades ago [4]. He formulated a personalised model for patient-care in which there would be mutual influence of the mind and body, in order to understand disease aetiology [5, 6].

Part of the challenge of implementing a comprehensive model of health lies in defining and modelling psychosocial determinants of health. Despite growing research in this field, there is no apparent consensus in the literature on a single definition of psychosocial health. In this study, we used the following WHO definition of mental health (2014) as a guiding principle: *“a state of wellbeing in which every individual realises their own potential, can cope with the normal stresses of everyday life*, *can* *work productively and fruitfully, and is able to make a contribution to their community.”* [7]. Specifically, we focused on the four highlighted components in order to identify and practically apply the psychosocial aspect of/to the model.

So far, despite much discussion there has been less focus on using individual-level data to formulate a practical model to guide clinical practice. In order to address this challenge, we have taken an empirical approach that is data-driven and exploratory, using data from a general birth cohort to translate this theoretical model into a practical framework that may be used to personalise preventative healthcare.

## Methods

### Study population and design

The study population comprised participants of the Northern Finland Birth Cohort 1966 (NFBC1966). It is an unselected, general population birth cohort including 96.3% of all births during 1966 in the two northern provinces of Finland, with clinical adult follow-ups at 31 and 46 years. The current analysis focuses on these follow-ups. The 31-year follow-up, conducted during 1997, consisted of a target population of 11,322 eligible individuals alive and living in Finland at this time (Supplementary 1). Of the target population, 77% of individuals completed the background questionnaire and 71% attended a clinical examination. The latest 46-year follow-up was conducted between April 2012 and February 2014. Of the target population consisting of 10,321 eligible individuals (Supplementary 1), 57% attended the clinical examination.

Individuals self-reporting a diabetes diagnosis at 31 years (*n* = 51) were excluded. A further 3,638 of the population did not participate in the 46-year clinical follow-up, and thus did not have an outcome measure. After exclusion, the  final study sample  comprised 5,078 participants (43% male) (Supplementary 1). The final sample included 103 participants who had not fasted before blood sample collection as statistical analyses showed no difference in laboratory measurements (*t*-test, *P* > 0.05) between these groups.

The study was approved by the Ethics Committee of the Northern Ostrobothnia Hospital District. All participants gave written informed consent.

### Biological and psychosocial variable selection

We used a systematic approach for bio-psychosocial indicator selection and applied the following criteria in the given order. Following an inventory of available variables within the NFBC1966 31-year data collection, we selected all those which fulfilled the following:

Criteria 1: A component of metabolic syndrome [8], consisting of measures relating to adiposity, insulin sensitivity, lipid levels and blood pressure, or an indicator of psychosocial health, based on the four components highlighted previously in the WHO (2014) definition; well-being, stresses of everyday health, work and community. We reviewed the data inventory and selected variables using our own judgement and *a priori* knowledge. Four members of the team independently reviewed the available data to ensure we did not miss any relevant variables.

Criteria 2: Easily obtainable as part of general routine health check-up. Table 1 shows the full variable list.

**Table 1:** Bio-psychosocial variables from 31-year data inventory according to selection criteria 1 & 2

Explanatory variables at age 31 years *n* = 5078	Descriptive statistics				Association with F-glucose at 46 years^a^		Inclusion
	*n*	Mean (SD) or number (%)	Min	Max	Estimate (beta, 95% CI)	*P* value	
Glucose (mmol/L)	3834	5.54 (0.59)	2.50^c^	21.20	0.41 (0.37, 0.45)	**<0.001**	✓
Insulin (µIU/mL)^b^	3818	7.40 (6.10–9.30)	2.90	72.10	0.04 (0.04, 0.05)	**<0.001**	✓
Waist circumference (cm)	3755	83.0 (11.7)	51.0	147.0	0.02 (0.02, 0.02)	**<0.001**	✓
BMI (kg/m^2^)	3883	24.4 (4.0)	15.3	54.4	0.05 (0.04, 0.05)	**<0.001**	✓
HDL-C (mmol/L)	3861	1.58 (0.38)	0.53	3.26	−0.41 (−0.48, −0.35)	**<0.001**	✓
TG (mmol/L)^b^	3861	0.97 (0.71–1.37)	0.19	11.10	0.22 (0.19, 0.26)	**<0.001**	✓
Systolic BP (mmHg)	3869	124.2 (13.5)	82.0	204.0	0.01 (0.01, 0.01)	**<0.001**	✓
Diastolic BP (mmHg)	3862	76.8 (11.5)	35.0	124.0	0.01 (0.01, 0.01)	**<0.001**	✓
Depression^b^	4969	1.27 (1.13–1.47)	1.00	3.80	−0.08 (−0.14, −0.01)	**0.017**	✓
Anxiety^b^	4980	1.20 (1.10–1.40)	1.00	3.50	−0.01 (−0.09, 0.06)	0.756	✗
Optimism	4973	19.1 (2.3)	6.0	30.0	−0.01 (−0.02, 0.00)	0.296	✗
Active coping	3338	14.3 (2.9)	5.0	20.0	−0.01 (−0.02, 0.00)	0.141	✗
Adaptive coping	3316	15.7 (3.3)	6.0	24.0	−0.02 (−0.03, −0.01)	**<0.001**	✓
Passive coping^b^	3386	6.0 (5.0–7.0)	4.0	15.0	0.00 (−0.01, 0.02)	0.797	✗
Basic education	5044						✓
Matriculation examination		2295 (45)			Ref		
Basic school		2749 (55)			0.14 (0.09, 0.18)	**<0.001**	
Further education	5012						✓
University		1362 (27)			Ref		
Vocational training		3403 (68)			0.07 (0.02, 0.12)	**0.008**	
No further education		247 (5)			0.19 (0.08, 0.30)	**0.001**	
Occupation	4960						✓
Professional		3143 (63)			Ref		
Manual worker/farmer		1560 (31)			0.17 (0.12, 0.22)	**<0.001**	
Unemployed		257 (5)			−0.04, (−0.15, 0.06)	0.406	
Household income	4354						✓
Rank 0 (highest)		818 (19)			Ref		
Rank 1		912 (21)			−0.01 (−0.09, 0.07)	0.770	
Rank 2		935 (21)			0.07 (−0.01, 0.15)	**0.071**	
Rank 3		876 (20)			0.01 (−0.07, 0.08)	0.897	
Rank 4 (lowest)		813 (19)			−0.01 (−0.09, 0.07)	0.780	
Marital status	5031						✓
Married/co-habiting		3779 (75)			Ref		
Single/divorced/widowed		1252 (25)			0.09 (0.04, 0.15)	**<0.001**	
Employment status	5000						✓
Employed		3526 (71)			Ref		
Not in labour force		909 (18)			−0.12 (−0.18, −0.07)	**<0.001**	
Unemployed		565 (11)			0.04 (−0.03, 0.11)	0.280	
Employment history	5023						✗
Mostly employed		4658 (93)			Ref	0.556	
Mostly unemployed		365 (7)			0.03 (−0.06, 0.11)		
Home ownership	5039						✓
Own home		2773 (55)			Ref		
Not own home		2266 (45)			0.04 (−0.00, 0.09)	**0.057**	
Sleep quality	5045						✓
Good		3389 (67)			Ref		
Medium		1368 (27)			0.02 (−0.03, 0.07)	0.513	
Bad		288 (6)			0.13 (0.03, 0.23)	**0.011**	
Life satisfaction	5027						✓
Very satisfied		1150 (23)			Ref		
Satisfied		3427 (68)			0.06 (0.00, 0.11)	**0.043**	
Average		335 (7)			0.19 (0.09, 0.29)	**<0.001**	
Not very satisfied		48 (1)			0.26 (0.03, 0.50)	**0.029**	
Not at all satisfied		67 (1)			0.12 (−0.08, 0.32)	0.250	

Mean and standard deviation (SD) are shown for all normally distributed continuous variables. *BMI* body mass index, *HDL-C* high density lipoprotein cholesterols, *TG* triglycerides, *SBP* systolic blood pressure, *DBP* diastolic blood pressure

^a^Mean fasting glucose at age 46 years was 5.51 (0.82) mmol/L, which was not statistically different to 31-year glucose when standardised to account for methodological differences in measurement (*P*  >  0.05)

^b^Median, 25 and 75% point estimate are presented for skewed continuous variables. Number and percentage (%) are shown for all categorical variables. All regression analyses are unadjusted

^c^One participant had a glucose level below 2.50 mmol/L, but was retained in the study

Criteria 3: Associated with the outcome, *i.e*. fasting plasma glucose (*P* < 0.10) at 46 years. The *F*-test determined inclusion of categorical variables (*P* < 0.10).

### Data collection

#### Metabolic variables at 31 years

Participants were invited to a clinical examination as described elsewhere [9]. Height and weight were measured to an accuracy of 0.1 cm and 0.1 kg, respectively, and converted to BMI (kg/m^2^). Waist circumference was measured from the point midway between the costal margin and iliac crest and recorded to an accuracy of 0.1 cm. Systolic and diastolic blood pressure (SBP and DBP) were measured twice with a mercury sphygmomanometer in sitting position from the right arm after 15 minutes of rest; two readings were taken and the average measurement used.

Laboratory samples were taken from participants. Analyses were conducted within 24 h for serum high density lipoproteins cholesterol (HDL-C) and triglycerides (TG) and determined by enzymatic methods using a Hitachi 911 Chemistry Analyser (Roche, Boehringer Mannheim, Germany). Blood samples for glucose assays were stored at −20 °C and analysed within 7 days of sampling by a glucose dehydrogenase method (Granutest 250, Diagnostica Merck, Darmstadt, Germany). Samples for assay of serum insulin were stored at −20 °C and analysed within 7 days of sampling using RIA (Pharmacia Diagnostics, Uppsala, Sweden) [9]. A correction constant was applied for participants having lipid or blood pressure altering medication (Supplementary 2) [10, 11].

#### Psychosocial indicators at 31 years

Information on psychosocial indicators was derived from a postal questionnaire. Questions related to well-being, stresses of everyday life, work and community. The full questions and response descriptions are available in Supplementary 3.

#### Outcome measure (fasting glucose) at 46 years

At 46 years, all participants attending the clinical examination followed similar protocol as before. Relevant to this study, blood samples were taken from participants after an overnight fast. Plasma samples were stored at −20 °C and analysed within 7 days of sampling for fasting plasma glucose (glucose dehydrogenase method; Advia 1800, Siemens Healthcare Diagnostics Inc.,Tarrytown, Ny, USA Country).

### Statistical analysis

#### Variable selection

**Fig. 1:**
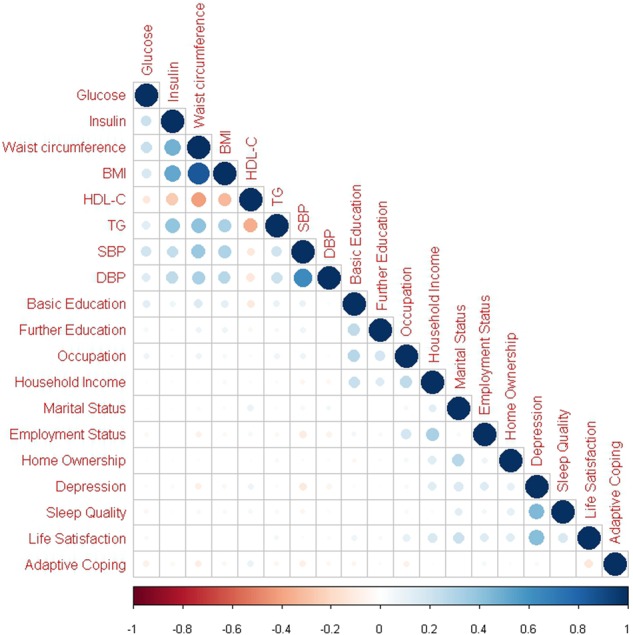
Correlation matrix of bio-psychosocial indicators. BMI body mass index, HDL-C high density lipoprotein cholesterol, TG, triglycerides, SBP systolic blood pressure, DBP diastolic blood pressure. Blue represents positive correlations and orange represents negative correlations. The size of dot reflects the strength of correlation

Descriptive statistics were generated for all explanatory variables and outcome measure and distributions were examined for normality. Univariate linear regression was used to assess association of each explanatory variable with the outcome of fasting plasma glucose at 46 years. A pearson’s correlation matrix was subsequently used to ensure factorability of the selected variables (Fig. 1). Males and females were analysed together as there were no major differences in factor loading patterns when stratified in preliminary analyses.

### Factor analysis

In all models, factor analysis was conducted using Mplus 7.0 [12]. Mplus uses full information maximum likelihood method to estimate the model parameters in order to account for missing data. [13].

Exploratory factor analysis (EFA) was conducted to identify the factor structure, followed by confirmatory factor analysis (CFA) to produce the final model. The dataset was halved using random number generation to perform cross-validation. There are no standardised guidelines for minimum sample size to be used for a valid EFA, although the general consensus is the larger the *N* and thus *N*:item ratio, the better [14]. Our sample size in the development half of the dataset is 2,556 reflecting a 134:1 participant:item ratio which is well above the 5:1 ratio suggested by some authors [15]. Geomin (oblique) rotations were used as it was hypothesised that the factors are likely to be correlated. Due to inclusion of categorical variables, the model parameters were estimated using mean-adjusted and variance-adjusted weighted least squares (WLSMV) method.

Optimum factor structure was selected based on a combination of model fit statistics, examination of scree plot and eigenvalues and inspection of factor loading patterns to ensure a scientifically feasible model [16]. At this stage, strength of factor loading was used to remove variables, which did not load significantly onto any factor or loaded with a score less than 0.3 onto any one factor. We used 0.3 as a cut-off based on our large sample size (*n* = 5,078) [17]. Model fit was assessed using Root Mean Square Error of Approximation (RMSEA), Comparative Fit Index (CFI) and Tucker Lewis Index (TLI) values. Commonly implemented guidelines of less than 0.08 for RMSEA and greater than 0.90 for CFI and TLI were used to assess model fit [18–20]. A chi-square test was also conducted to evaluate the congruency between the hypothesised model and empirical evidence, although it is well recognised that chi-square tests are sensitive to large sample size [18, 21].

Factor scores were extracted and used in multivariable linear regression models to assess associations and interrelationships between factor scores and fasting glucose. These models were adjusted for sex and each of the other factors using a forward stepwise approach. The lowest AIC was primarily used to determine the best prediction model, however we also considered *R*^2^ and BIC.

## Results

### Variable selection

Following data inventory, 26 variables relating to bio-psychosocial health were selected. We excluded social anhedonia according to criteria 2 [22, 23]. Of the remaining 25 variables (Table 1), we excluded employment history, anxiety, functioning, optimism, active and passive coping as they did not associate with fasting glucose at 46 years (criteria 3).

Distinct correlation clusters were visible, highlighting groups of biological and psychosocial related variables (Fig. 1). The strongest correlations were present between the cardio-metabolic variables.

### Bio-psychosocial factors

A base of 19 items relating to bio-psychosocial health were entered into an EFA using the testing half of the dataset (*n* = 2,556). Five eigenvalues were greater than one, and therefore we tested models with a one-factor to five-factor structure. Examination of the scree plot (Supplementary 4) suggested that a three, four or five-factor structure fitted best, although model fit statistics tended to favour the four or five-factor model (Table 2). These factors also showed consistency with the patterns observed in the correlation matrix (Fig. 1) and appeared to separate into biological, socioeconomic and psychosocial latent factors. As the number of factors increased, the biological variables separated into metabolic and blood pressure factors and psychosocial separated into a psychological and additional (psycho)social factor.

**Table 2:** EFA of bio-psychosocial variables at 31 years associated with fasting glucose at 46 years (n = 2,556)

	Three-factor model			Four-factor model				Five-factor model				
Model fit	*χ*2 = 2228.67, df = 117, *P* < 0.001; CFI = 0.91; TLI = 0.87; RMSEA = 0.084			*χ*2 = 1530.82, df = 101, *P* < 0.001; CFI = 0.94; TLI = 0.90; RMSEA = 0.074				*χ*2 = 934.98, df = 86, *P* < 0.001; CFI = 0.96; TLI = 0.91; RMSEA = 0.062				
**Variables**	**1**	**2**	**3**	**1**	**2**	**3**	**4**	**1**	**2**	**3**	**4**	**5**
Insulin	0.156	**0.563**		0.254	**0.557**		−0.076	0.062	**0.441**	**−** **0.441**		0.233
Waist circumference		**0.818**			**0.752**	0.131			**0.860**		0.069	−0.075
BMI	−0.097	**0.813**	0.069	−0.048	**0.796**		0.053	-0.069	**0.937**	0.039	−0.049	
SBP	0.238	**0.420**	−0.146		0.039	**0.854**	−0.058				**0.867**	−0.084
DBP	0.113	**0.425**	−0.059	−0.079	0.150	**0.644**	0.024	−0.108	0.104		**0.673**	0.038
HDL-C	−0.042	−**0.454**		−0.086	**−0.449**			−0.093	**−** **0.412**	0.071		
TG		**0.564**	0.067	0.086	**0.557**				**0.417**	−0.244		0.186
Glucose	**0.376**	0.200	−0.103	0.259	0.129	0.138	−0.077	0.206	0.066	−0.180	0.151	
Basic education	**0.722**	0.094		**0.737**	0.120		−0.027	**0.807**				−0.042
Further education	**0.456**			**0.493**				**0.526**				
Occupation type	**0.503**		0.193	**0.536**			0.159	**0.552**		0.143		
Income	**0.383**		**0.318**	**0.453**	−0.063		0.283	**0.450**	0.048	**0.468**		
Employment status	0.115	−0.111	**0.393**	0.219	−0.067		**0.354**	0.252		**0.376**	−0.074	0.133
Marital status		0.073	**0.460**	0.043	−0.122	**0.348**	**0.490**			**0.355**	**0.302**	**0.326**
Home ownership			**0.392**			0.145	**0.389**		0.068	**0.360**	0.085	0.227
Depression	−0.190		**0.720**	−0.101	0.076	−0.064	**0.717**				−0.116	**0.750**
Sleep quality	−0.193		**0.567**	−0.126	0.120	−0.075	**0.560**	-0.061		−0.066		**0.599**
Life satisfaction		0.082	**0.638**	0.051		0.193	**0.655**	0.085		0.208	0.156	**0.551**
Adaptive coping	−0.070	−0.097				−0.096					−0.106	

Model fit statistics and geomin factor loadings for 3–5 factor structures. Results shown are for the testing half of the dataset. Empty squares represent insignificant loadings. All squares outlined in bold, containing numbers in bold represent loadings greater than 0.3. Model fit statistics: *χ*2 chi-square, *df* degrees of freedom, *CFI* comparative fit index, *TLI* Tucker Lewis index, *RMSEA* root mean square error of approximation

*BMI* body mass index, *HDL-C* high density lipoprotein cholesterols, *TG* triglycerides, *SBP* systolic blood pressure, *DBP* diastolic blood pressure

In all three models, variables loaded strongly to their factor. We then looked for a clean factor structure, i.e. no cross-loadings, and scientific plausibility [14]. The five-factor structure was excluded due to strong cross-loading of insulin and marital status, and difficulty in scientifically identifying the additional (psycho)social factor (Supplementary 7). Two items (glucose and adaptive coping) were excluded from the final model due to loading below 0.3 to any one factor (Table 2).

Factors were named to reflect their included variables. Basic and further education, occupation and household income loaded onto the first factor named *‘socioeconomic’* and accounted for 19% of the total variance of the model. Insulin, waist circumference, BMI, HDL-C and TG loaded onto a *‘metabolic’* factor and accounted for 14% of variation. Marital status, employment status, home ownership, depression, sleep quality and life satisfaction accounted for 9% of variation and was named the *‘psychosocial’* factor. SBP and DBP loaded onto a *‘blood pressure’* factor and accounted for 7% of variation (Supplementary 4).

**Fig. 2:**
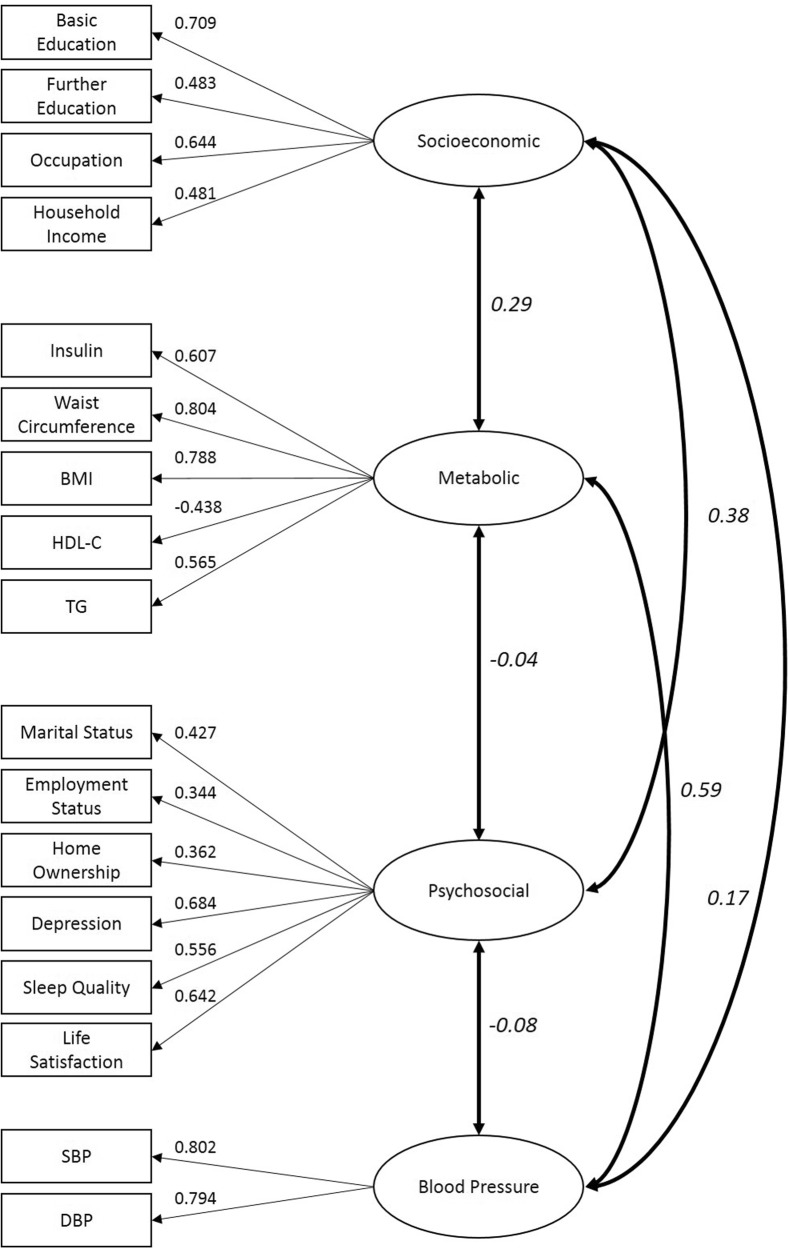
Confirmatory factor analysis of four-factor structure containing the bio-psychosocial indicators. BMI body mass index, HDL-C high density lipoprotein cholesterol, TG triglycerides, SBP systolic blood pressure, DBP diastolic blood pressure. Boxes represent observed indicators, circles represent latent factors and two-way arrows represent correlation between factors. Pearson correlation coefficients are written in italics. *χ*2 = 2510.83, df = 113, *N* = 5 078, *P* < 0.01; CFI = 0.92, RMSEA = 0.065

This model was replicated in the other half of the dataset and CFA was then performed using the full dataset. This demonstrated a good fit for the data (RMSEA = 0.065; CFI = 0.92; TLI = 0.90) and was chosen as the final model (Fig. 2).

### Sensitivity analysis

EFA was also conducted in the full sample and demonstrated similar model fit, strength of loading and loading patterns (Supplementary 5). Additionally, CFAs were conducted for all three of the potential factor structures to examine model fit and strength of loading of each variable. This confirmed the four-factor structure was optimal (Supplementary 6 & 7).

### Predicting fasting glucose from factors scores

Table 3 shows univariable results for associations between each factor and fasting glucose at 46 years (model 1). The socioeconomic, metabolic and blood pressure factors were all associated (*P* < 0.05) with fasting glucose, and this remained following sex adjustment (Model 2). The psychosocial factor, however, was not associated with fasting glucose at 46 years until it was adjusted for sex.

**Table 3:** Association of 31-year factor scores with fasting glucose at 46 years

	Model 1			Model 2			Model 3		
	Estimate (beta, 95% CI)	*P* value	*R* ^2^	Estimate (beta, 95% CI)	*P* value	R^2^	Estimate (beta, 95% CI)	*P* value	*R* ^2^
Socioeconomic	0.178 (0.136, 0.219)	**<0.001**	0.014	0.116 (0.075, 0.158)	**<0.001**	0.067	0.011 (-0.035, 0.057)	0.652	
Metabolic	0.112 (0.101, 0.123)	**<0.001**	0.076	0.090 (0.079, 0.101)	**<0.001**	0.105	0.087 (0.073, 0.100)	**<0.001**	
Psychosocial	0.042 (-0.022, 0.019)	0.201	<0.001	0.081 (0.019, 0.144)	**0.011**	0.062	0.086 (0.018, 0.153)	**0.013**	
Blood Pressure	0.018 (0.016, 0.021)	**<0.001**	0.038	0.012 (0.009, 0.015)	**<0.001**	0.076	0.001 (-0.002, 0.004)	0.480	0.107

Model 1: unadjusted; model 2: adjusted for sex; model 3: adjusted for sex and all other factors

*SE* standard error, *CI* confidence intervals, beta = increase in fasting glucose at 46 years by one unit increase in factor score

**Fig. 3:**
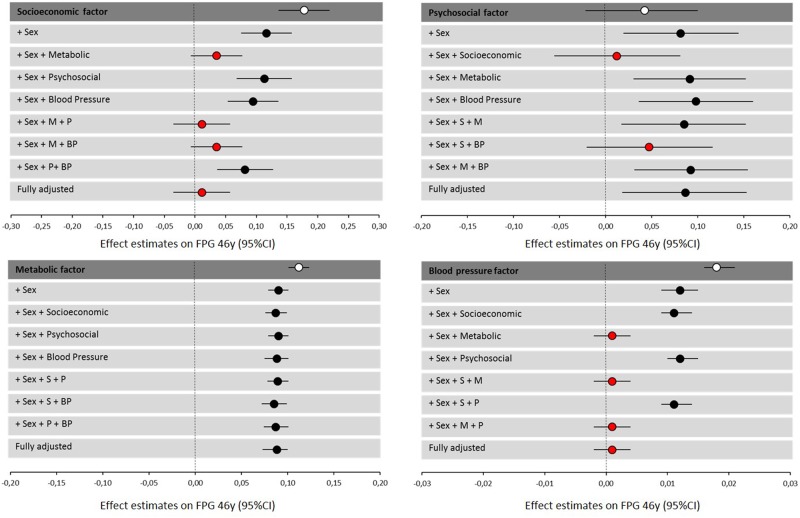
Forest plots showing the effect estimates (beta, 95% CI) of each factor on fasting plasma glucose at age 46 years. Each factor has been sequentially adjusted for sex and the other factors. *S* socioeconomic factor, *M* metabolic factor, *P* psychosocial factor, *BP* blood pressure factor

Multivariable regression analysis assessed whether associations remained significant when additionally adjusted for the other factors (model 3) and the forest plots show their stepwise addition (Fig. 3). The metabolic factor appeared to have the most robust effect on fasting glucose. Unsurprisingly, the metabolic factor subsequently had a large influence on the association of the other factors with fasting glucose. Although the socioeconomic factor initially had the largest effect, it was completely attenuated by the addition of the metabolic factor. This was also the case for blood pressure. Noticeably, the psychosocial effect on fasting glucose at 46 years was only attenuated by the socioeconomic factor.

Of the 11 possible factor combinations (Supplementary 8), the best prediction model for fasting glucose at 46 years was the combination of metabolic and psychosocial factors when adjusted for sex. This explained 10.7% of the variation in fasting glucose.

### Attrition analysis

There were 5,641 participants with a recorded fasting glucose measurement at age 46 years. However, 563 of these participants did not partake in the 31-year follow-up and thus were not included in this study. These participants were more likely to be male (55%; chi-sq < 0.05) and tended to have higher fasting glucose at 46 years (5.65 vs 5.50 mmol/L; *P* < 0.05).

## Discussion

Using a systematic variable selection strategy, we derived four latent factors at age 31 years underlying fasting plasma glucose in midlife. These factors delineated socioeconomic, metabolic, psychosocial and blood pressure components and were named accordingly. Analyses on their effect size against fasting glucose 15 years later in life brought additional insights on the nature of these associations. It particularly highlighted independent effects for the metabolic and psychosocial factors.

### Latent factors

EFA showed distinct variable clusters similar to biological and psychosocial groupings observed in the correlation matrix (Fig. 1). The fit statistics and general structure were consistent in both the full dataset and random half used for cross-validation. Minor differences were observed only in the loading of glucose to the socioeconomic factor in the three-factor structure and the additional cross-loading of marital status to the psychological factor in the five-factor structure (Supplementary 5).

Metabolic syndrome is characterised by simultaneous observation of metabolic abnormalities including abdominal obesity, hypertension, hyperglycaemia and dyslipidemia [24, 25]. Previous studies attempting to capture the factorial structure of metabolic syndrome used an a-priori driven CFA that enforced each of the metabolic syndrome components onto four first order factors (adiposity, insulin resistance, hypertension, dyslipidaemia) before loading onto an overall metabolic factor [26, 27]. Although their model demonstrated good fit according to standard criteria, glucose loaded weakly onto the insulin resistance factor, and hypertension had the weakest loading onto the overall metabolic factor, suggesting that the model may be improved. We observed similar patterns of results in our factor structure; initially the blood pressure variables clustered with the metabolic components. However, we discovered a better fit for the data was observed when these separated into an individual blood pressure factor. Although the variables in the present approach were selected using predetermined generic criteria, the procedure was data-driven. This allowed us the opportunity to observe the best fit for our data without enforcing a structure based only on a-priori categorisation of metabolic syndrome. It may be surprising that glucose at 31 years was excluded from our final model. It did not load strongly to any one factor, but loaded significantly onto every factor. This is not unexpected as variables were selected based on their association with fasting glucose at 46 years. It is also interesting that as the factors separated further, glucose shows a higher affinity towards the socioeconomic factor than the biological as we may expect. This is more evident in the testing dataset results (Table 2), but can also be observed in the full dataset, particularly within the five-factor model (Supplementary 5).

In terms of the less understood non-biological factors, using a data-driven, exploratory approach allowed us to elucidate factors consisting of variables that shared some commonality. It helped differentiate between those variables more representative of socioeconomic position, and variables, which we believe relate more to psychosocial health.

Basic and further education, occupation and household income clustered together to form the socioeconomic factor. Although these measures seem similar, they all capture slightly different pathways in which health may be influenced. Income provides access to resources, which enables greater options in food choices, a higher level of health care and more physical activity possibilities [28]. Education provides increased awareness of health issues and a greater willingness to engage in healthy behaviours [29]. Occupation may determine flexibility in working hours and schedule, thus allowing available time to engage in leisure and exercise. Additionally, worksite health promotion programmes and policies that protect occupational safety may also play a role [28]. Traditionally researchers have used only one of these measures to study socioeconomic position, but the use of a composite factor may reflect many complex differences in social health.

Marital status, home ownership, employment status, depression, sleep quality and life satisfaction loaded onto another factor, which we have termed psychosocial. Martikainen *et al* [30] has suggested a working definition of psychosocial determinants of health as “*pertaining to the influence of social factors on an individual’s mind or to the interrelation of behavioural and social factors*”. The implication of this definition is that psychosocial factors in the context of health research can be viewed as mediating effects of socioeconomic structural factors on individual health outcomes or conditioned and modified by the socioeconomic structures in which they exist.

### Predicting fasting glucose from factors scores

The metabolic factor demonstrated the most robust and stable association with glucose at age 46 following multiple adjustments. The blood pressure factor was also significantly associated with fasting glucose both unadjusted and adjusted for sex. However, when additionally adjusted for all other factors, its contribution to the model was no longer observed. We noted that it is specifically the addition of the metabolic factor which attenuates this relationship, indicating that the effects of blood pressure on later fasting glucose may be mostly mediated by metabolic components. Nonetheless, despite the separation of blood pressure to form a separate factor from the metabolic factor, we observed that they were still highly correlated (pearson correlation coefficient = 0.59; Fig. 2). We observed an especially strong correlation structure between blood pressure and the insulin-WC-BMI cluster (Fig. 1) that supports the hypothesis of a common origin of these biological functions [31]. There seems to be shared molecular architecture, probably originating from foetal development that links these functions together with the regulation of fasting glucose [31]. However, knowledge gaps remain to delineate the causal mechanisms and their effect on the regulation of glycemic health.

The socioeconomic factor also demonstrated a significant association with fasting glucose, which persisted following adjustment for sex but not adjustment for all other factors. Looking closer at the stepwise addition of factors to the socioeconomic factor, it appears that it is only the metabolic factor which attenuates its effect. This suggests that some of its influence may be explained, at least in part, by health behaviours acting on metabolic processes. This is consistent with findings of recent publications demonstrating relationships between socioeconomic position and cardio-metabolic health outcomes [32–35]. Health behaviours such as alcohol consumption, smoking status, dietary intake and physical activity directly impact metabolic status, particularly adiposity and lipid levels. In the present study we did not use lifestyle information as it is difficult to obtain objective measurements, especially in routine health care. However, we believe lifestyle is captured within this socioeconomic factor as it is frequently reported [32, 33] and can be included in further models to investigate the effects of modifiable factors.

The effect of the psychosocial factor was in contrast to the others. No association with glucose was observed when unadjusted. However, sex-adjustment and adjustment for all other factors, showed a significant association which was not even diminished by the strong effects of the metabolic factor. Closer examination of the stepwise sex-adjusted model showed that only the addition of the socioeconomic factor attenuated the psychosocial relationship with glucose. Psychosocial and socioeconomic factors are very closely related and appear to be linked by household income as seen in cross-loadings from EFA (Table 2) and correlation matrix (Fig. 1). Thus, it is expected that their effects on the outcome are also closely related and may act via similar pathways. We observed no significant sex differences in the formation of the factors, however, a larger sample size would allow sex-specific analysis and may help to identify what is causing this factor to behave differently in males and females.

We speculate that the psychosocial factor may actually be capturing a ‘stress’ effect. Strong biological links exist between glucose metabolism and neuroendocrine responses to variation in psychosocial well-being. The hormonal outputs of the hypothalamic-pituitary-adrenal axis, such as the glucocorticoids, acutely alter short-term glucose metabolism [36]. Long-term exposure to psychosocial stressors may contribute to chronic glycaemic dysregulation in individuals. The independent effects of the psychosocial and metabolic factors suggest that there are separate biological pathways in which fasting glucose levels are maintained [37–40].

### Strengths and limitations

We used a large, unselected birth cohort that is particularly rich in data during these two follow-up periods. At 31 and 46 years, the participants are still young, and therefore less likely to experience acceleration in glucose associated with T2D or its pre-clinical stages. However, we acknowledge that more frequent follow-ups would allow us to identify participants with high risk of developing T2D.

We recognise that our study does have some limitations. As with all longitudinal designs there is missing data and this may be partly due to attrition. We conducted attrition analysis in order to take this into consideration, and we aimed to overcome the missing at random data by using Mplus software, which compensates for missing data. We have tried to be as descriptive as possible in naming the latent factors, however, it is challenging to accurately capture the trait they are representing.

There is always a trade-off between incorporating the maximum available dimensions to produce a completely comprehensive model, and using widely available and easily accessible measures to achieve similar results which is what we have been aiming to do in this study. For example we did not include lifestyle variables as they are time-consuming to analyse in a meaningful manner and are likely to already be captured via the socioeconomic factor. However, we have demonstrated reasonably good model fit statistics for all models, which can be explained scientifically and we have used cross-validation techniques to further strengthen design.

### Implications

This study is the first step in developing a model, which may be used clinically to identify those with an increased risk of developing poor glycaemic health and T2D. Early identification of these individuals can provide an opportunity to implement targeted interventions and policy recommendations for personalised prevention. The following steps as part of the DynaHEALTH project will aim to translate this systematic approach to create risk scores during the life course to reflect the dynamic and trajectory of deteriorating glycaemic control.

### Conclusions

The present study supports evidence for the bio-psychosocial nature of adult glycemic health and utilises an example to understand and reduce its complexity. To date, most studies have attempted to analyse the biological and psychological factors separately, making it difficult to distinguish the relations of these components. However, this is critical in the context of complex life-long, non-communicable diseases such as T2D where physiological and social functioning are impacted. Here we reported a systematic data-driven approach to study the relationship between the factors associated with the maintenance of normal fasting glucose. The methodology employed brings transparency in variable selection and is easily transferable to other traits and complex diseases with strong interplay between biological and psychosocial factors.

## Electronic supplementary material

Supplementary Legends

Supplementary Figure 1

Supplementary Table 2

Supplementary Table 3

Supplementary Figure 4

Supplementary Table 5

Supplementary Figure 6

Supplementary Figure 7

Supplementary Table 8

## References

[1] WHO Global Report on Diabetes 2016.

[2] Danaei G, Finucane M, Lu Y, Singh G, Cowan M, Paciorek C (2011). National, regional and global trends in fasting plasma glucose and diabetes prevalence since 1980: systematic analysis of health examination surveys and epidemiological studies with 370 country-years and 2·7 million participants. Lancet.

[3] Tabák AG, Jokela M, Akbaraly TN, Brunner EJ, Kivimäki M, Witte DR (2009). Trajectories of glycaemia, insulin sensitivity, and insulin secretion before diagnosis of type 2 diabetes: an analysis from the Whitehall II study. Lancet.

[4] Engel GL (1977). The need for a new medical model: a challenge for biomedicine. Science.

[5] Fava G, Sonino N (2017). From the lesson of George Engel to current knowledge: the bio-pschyosocial model 40 years later. Psychother Psychosom.

[6] Young-Hyman D, de Groot M, Hill-Briggs F, Gonzalez JS, Hood K, Peyrot M (2016). Psychosocial care for people with diabetes: a position statement of the American diabetes association. Diabetes Care.

[7] WHO Mental Health 2014 Available from: http://www.who.int/features/factfiles/mental_health/en/ Cited 18 Dec 2017.

[8] International Diabetes Federation. Metabolic Syndrome. 2006.

[9] Sovio U, Kaakinen M, Tzoulaki I, Ruokonen A, Pouta A, Hartikainen AL (2014). How do changes in body mass index in infancy and childhood associate with cardiometabolic profile in adulthood? Findings from the Northern Finland Birth Cohort 1966 Study. Int J Obes.

[10] Tobin MD, Sheehan NA, Scurrah KJ, Burton PR (2005). Adjusting for treatment effects in studies of quantitative traits: antihypertensive therapy and systolic blood pressure. Stat Med.

[11] Wu J, Province MA, Coon H, Hunt S, Eckfeldt J, Arnett D (2007). An investigation of the effects of lipid-lowering medications: genome-wide linkage analysis of lipids in the HyperGEN study. BMC Genet.

[12] Muthèn L, Muthèn B. Mplus user’s guide. 4th ed. Los Angeles, CA: Muthèn & Muthèn; 1998–2007..

[13] Kenward M, Molenberghs G (1998). Likelihood based frequentist inference when data are missing at random. Stat Sci.

[14] Osborne JW, Costello AB. Sample size and subject to item ratio in principal components analysis. PARE. 2004;**9**.

[15] Sapnas KG, Zeller RA (2002). Minimizing sample size when using exploratory factor analysis for measurement. J Nurs Meas.

[16] Velicer W, Jackson D (1990). Component analysis versus common factor-analysis—some further observations. Multivar Behav Res.

[17] Hair JF, Tatham RL, Anderson RE, Black W (1998). Multivariate data analysis.

[18] Bentler PM (1990). Comparative fit indexes in structural models. Psychol Bull.

[19] Browne M, Cudeck R, Bollen KA, Long JS (1993). Alternative ways of assessing model fit. Testing structural equation models.

[20] Hu L & Bentler P. Cutoff criteria for fit indexes in covariance structure analysis: conventional criteria versus new alternatives. Structural Equation Modeling. 1999. pp. 1–55.

[21] McDonald RP, Marsh HW (1990). Choosing a multivariate model: Noncentrality and goodness of fit. Psychol Bull.

[22] Chapman LJ, Chapman JP, Kwapil TR, Eckblad M, Zinser MC (1994). Putatively psychosis-prone subjects 10 years later. J Abnorm Psychol.

[23] Kwapil TR (1998). Social anhedonia as a predictor of the development of schizophrenia–spectrum disorders. J Abnorm Psychol.

[24] Eckel RH, Grundy SM, Zimmet PZ (2005). The metabolic syndrome. Lancet (Lond, Engl).

[25] Grundy SM, Cleeman JI, Daniels SR, Donato KA, Eckel RH, Franklin BA (2005). Diagnosis and management of the metabolic syndrome: an American Heart Association/National Heart, Lung, and Blood Institute Scientific Statement. Circulation.

[26] Marsland AL, McCaffery JM, Muldoon MF, Manuck SB (2010). Systemic inflammation and the metabolic syndrome among middle-aged community volunteers. Metabolism.

[27] McCaffery J, Marsland A, Strohacker K, Muldoon M, Manuck S. Factor structure underlying components of allostatic load. PLoS ONE. 2012;**7** (10). 10.1371/journal.pone.0047246 PMC3480389 23112812

[28] Kaplan R, Spittel M, David D (Eds). Population health: behavioral and social science insights. AHRQ Publication No. 15-0002. Rockville, MD: Agency for Healthcare Research and Quality and Office of Behavioral and Social Sciences Research, National Institutes of Health; July 2015.

[29] Cutler DM, Lleras-Muney A (2010). Understanding differences in health behaviors by education. J Health Econ.

[30] Martikainen P, Bartley M, Lahelma E (2002). Psychosocial determinants of health in social epidemiology. Int J Epidemiol.

[31] Horikoshi M, Beaumont R, Day FR, Warrington NM, Kooijman MN, Fernandez-Tajes J (2016). Genome-wide associations for birth weight and correlations with adult disease. Nature.

[32] Stringhini S, Dugravot A, Shipley M, Goldberg M, Zins M, Kivimäki M, et al. Health behaviours, socioeconomic status, and mortality: further analyses of the British Whitehall II and the French GAZEL Prospective Cohorts. PLoS Medicine. 2011;8 (2). 10.1371/journal.pmed.1000419 PMC3043001 21364974

[33] Stringhini S, Tabak A, Akbaraly T, Sabia S, Shipley M, Marmot M, et al. Contribution of modifiable risk factors to social inequalities in type 2 diabetes: prospective Whitehall II cohort study. *BMJ*. 2012;345 :e5452. 10.1136/bmj.e5452 PMC3424226 22915665

[34] Paek KW, Chun KH, Jin KN, Lee KS (2006). Do health behaviours moderate the effect of socioeconomic status on metabolic syndrome?. AEP.

[35] Pampel F, Krueger P, Denney J (2010). Socioeconomic disparities in health behaviors. Annu Rev Sociol.

[36] Herman JP, McKlveen JM, Solomon MB, Carvalho-Netto E, Myers B (2012). Neural regulation of the stress response: glucocorticoid feedback mechanisms. Braz J Med Biol Red.

[37] Derogatis Lipman RS, Rickels K, Uhlenhuth EH, Covi L (1974). *The Hopkins Symptom Checklist (HSCL): a self-report symptom inventory*. Behav Sci.

[38] Sintonen H. The 15-D measure of health related quality of life: reliability, validity and sensitivity of its health state descriptive system. Centre for Health Program Evaluation, working paper 41. 1994. ISSN 1038–9547.

[39] Ek E, Koiranen M, Raatikka VP, Järvelin MR, Taanila A (2008). Psychosocial factors as mediators between migration and subjective well-being among young Finnish adults. Soc Sci Med.

[40] OECD. Divided we stand—why inequality keeps rising, Paris. ISBN: 9789264111639 OECD Publishing 400 pages, 2011

