# Supplementary material for: Understanding the complexity of glycaemic health: systematic bio-psychosocial modelling of fasting glucose in middle-age adults; a DynaHEALTH study
Source: Int J Obes (Lond). 2018 Aug 17;43(6):1181–92. doi: 10.1038/s41366-018-0175-1 (PMC6760581; doi:10.1038/s41366-018-0175-1)
Supplement: Supplementary file 1 — Supplementary Legends [file 41366_2018_175_MOESM1_ESM.docx]

**Supporting Information Captions**

**S1.jpg** - Flow chart of NFBC 1966 participant selection for inclusion in final study population.

**S2.docx** - Correction constant for lipid and blood pressure medication.

**S3_Revised.docx** - Variable descriptions detailing methods of collection, analytical procedures, original and modified coding of categorical variables.

**S4.jpg** - Scree plot for exploratory factor analysis demonstrating five factors with an eigenvalue greater than one. Based on the eigenvalue (the dashed line indicates an eigenvalue of 1.0) and scree plot criteria, a three-, four-, or five-factor solution would be acceptable. Subjective examination based on interpretability and simple structure led the authors to use a four-factor solution.

**S5.docx** - Exploratory Factor Analysis for all bio-psychosocial variables at 31 years which were significantly associated with fasting glucose at 46 years (n=5078).

**S6_Revised.jpg** - Confirmatory factor analysis of three-factor structure containing the bio-psychosocial indicators.

BMI, body mass index; HDL-C, high density lipoprotein cholesterols; TG, triglycerides; SBP, systolic blood pressure; DBP, diastolic blood pressure. Boxes represent observed indicators and circles represent latent factors. Factor loadings are presented for each variable. χ2 = 3300.90, df = 116, N = 5 078, p < 0.01; CFI =0.89, RMSEA =0.074.

**S7_Revised.jpg** - Confirmatory factor analysis of five-factor structure containing the bio-psychosocial indicators.

BMI, body mass index; HDL-C, high density lipoprotein cholesterols; TG, triglycerides; SBP, systolic blood pressure; DBP, diastolic blood pressure. Boxes represent observed indicators and circles represent latent factors. Factor loadings are presented for each variable. χ2 = 2302.51, df = 109, N = 5 078, p < 0.01; CFI =0.92, RMSEA =0.063.

**S8.docx**: Association of 31-year factor scores with F-Glucose at 46 years (Model 1: unadjusted for sex; Model 2: adjusted for sex).
