# Supplementary figures and images for: Understanding the complexity of glycaemic health: systematic bio-psychosocial modelling of fasting glucose in middle-age adults; a DynaHEALTH study
Source: Int J Obes (Lond). 2018 Aug 17;43(6):1181–92. doi: 10.1038/s41366-018-0175-1 (PMC6760581; doi:10.1038/s41366-018-0175-1)

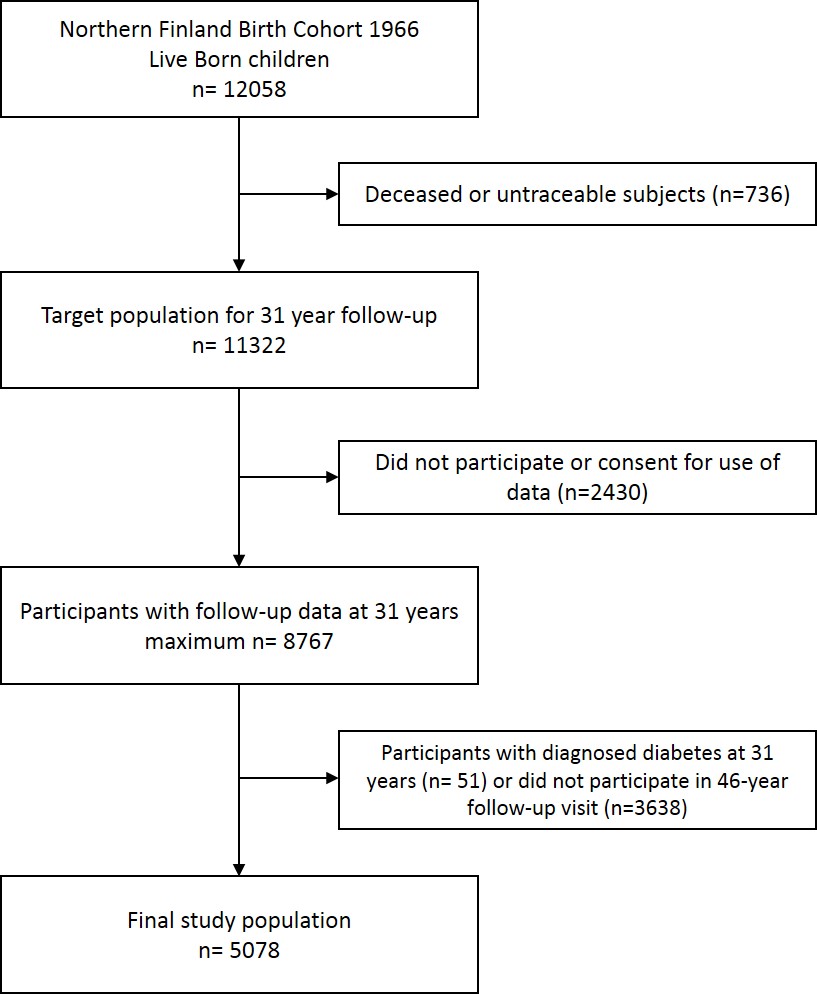

Supplement: Supplementary file 2 — Supplementary Figure 1 [file 41366_2018_175_MOESM2_ESM.jpg]

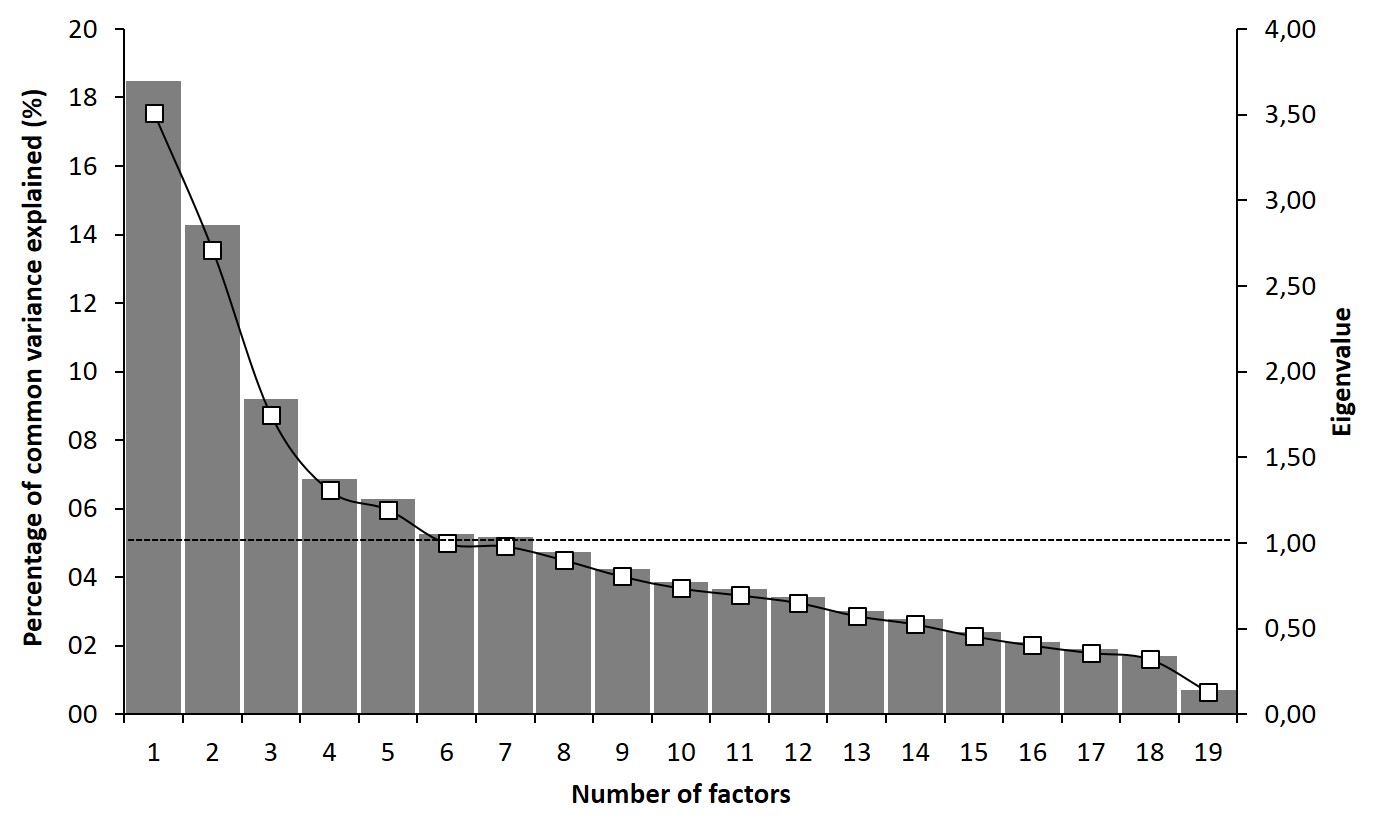

Supplement: Supplementary file 5 — Supplementary Figure 4 [file 41366_2018_175_MOESM5_ESM.jpg]

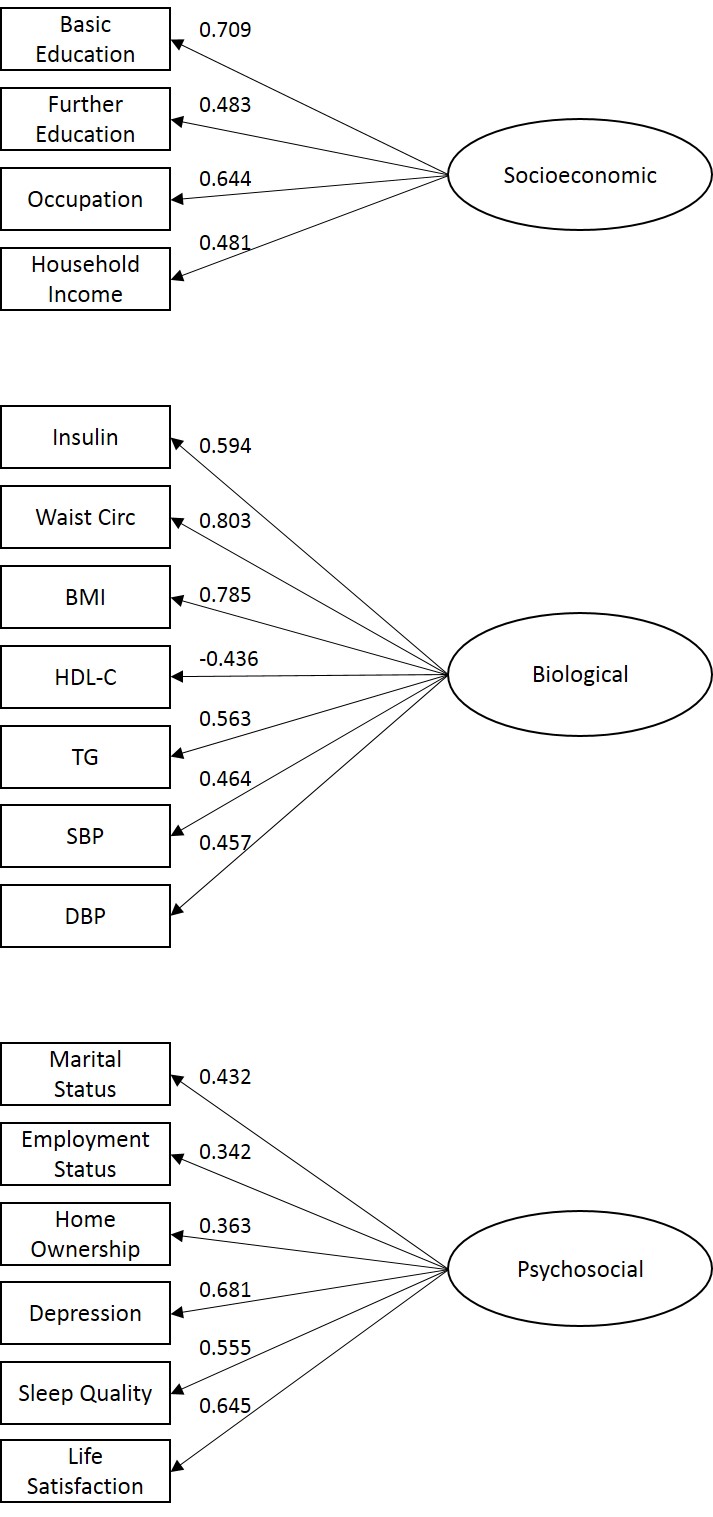

Supplement: Supplementary file 7 — Supplementary Figure 6 [file 41366_2018_175_MOESM7_ESM.jpg]

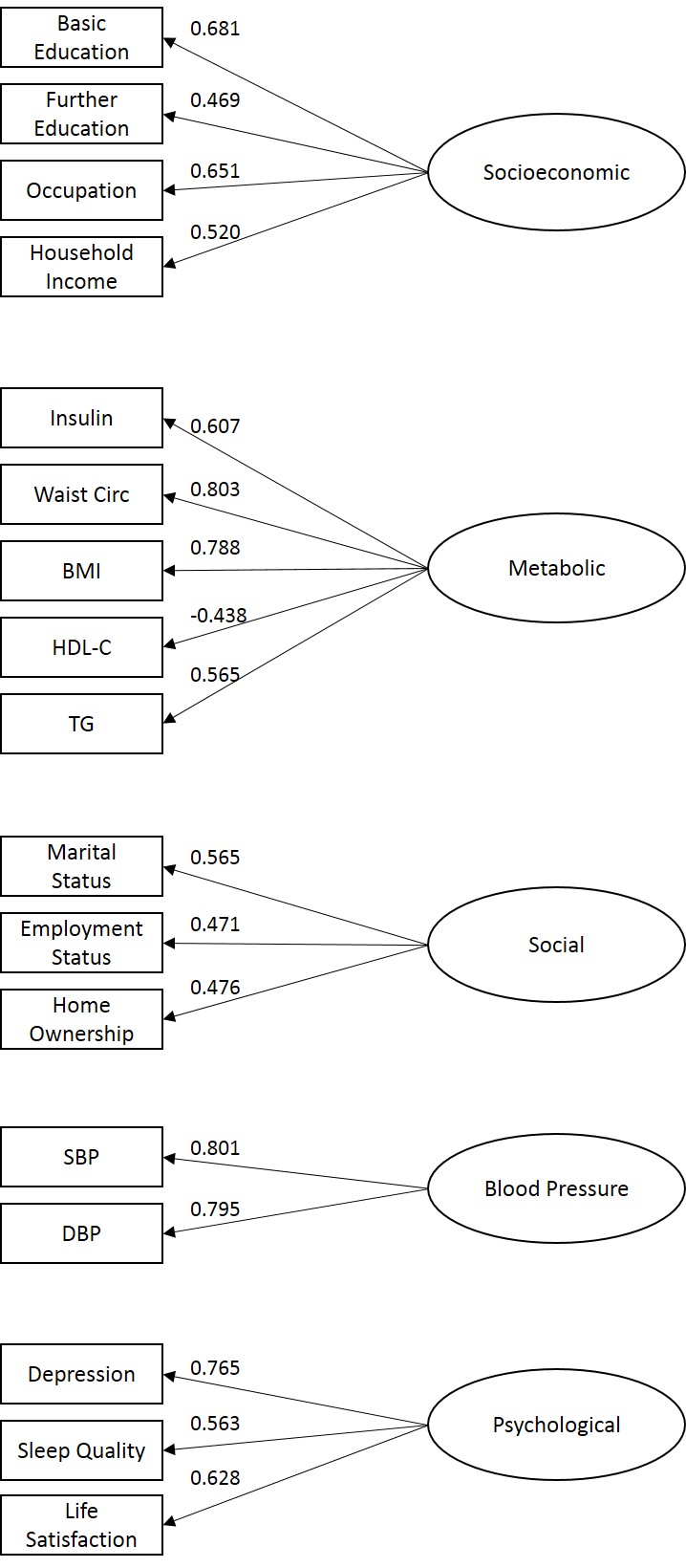

Supplement: Supplementary file 8 — Supplementary Figure 7 [file 41366_2018_175_MOESM8_ESM.jpg]
