# Supplementary material for: Understanding the complexity of glycaemic health: systematic bio-psychosocial modelling of fasting glucose in middle-age adults; a DynaHEALTH study
Source: Int J Obes (Lond). 2018 Aug 17;43(6):1181–92. doi: 10.1038/s41366-018-0175-1 (PMC6760581; doi:10.1038/s41366-018-0175-1)
Supplement: Supplementary file 3 — Supplementary Table 2 [file 41366_2018_175_MOESM3_ESM.docx]

**S2: Correction constant for lipid and blood pressure medication.**

| **Correction constant for lipid medication** | | | |
| --- | --- | --- | --- |
|  |  | mg/dl | mmol/l |
| HMG-CoA reductase inhibitors (statins) | TG | + 18.4 | + 0.208 |
|  | HDL | - 2.3 | - 0.06 |
| Fibrates | TG | +57.1 | + 0.645 |
|  | HDL | -5.9 | -0.153 |
| Bile acid sequestrants | TG | + 0 | + 0 |
|  | HDL | -1.9 | -0.049 |
| **Correction constant for blood pressure** | | | |
|  |  | mmHg |  |
| anti-hypertensive or blood pressure lowering medication | SBP | + 15 |  |
|  | DBP | + 10 |  |
|  | | | |
