# Supplementary material for: Understanding the complexity of glycaemic health: systematic bio-psychosocial modelling of fasting glucose in middle-age adults; a DynaHEALTH study
Source: Int J Obes (Lond). 2018 Aug 17;43(6):1181–92. doi: 10.1038/s41366-018-0175-1 (PMC6760581; doi:10.1038/s41366-018-0175-1)
Supplement: Supplementary file 4 — Supplementary Table 3 [file 41366_2018_175_MOESM4_ESM.docx]

**S3: Variable descriptions detailing methods of collection, analytical procedures, original and modified coding of categorical variables.**

| Data Collection | Variable | Type | Method of Measurement |
| --- | --- | --- | --- |
| Clinical Examination | **Blood Pressure (systolic and diastolic)** | Continuous | Blood pressure (SBP, DBP mmHg) was measured in a sitting position after 15 min of rest, from the right upper arm using an OMRON blood pressure monitor (OMRON Matsusaka Co. Ltd, Japan). The appropriate cuff size was determined by the subject’s arm circumference. Two readings were taken 2 min apart, and the average of the measurements was used. |
|  | **Body Mass Index (BMI)** | Continuous | Weight (kg) and height (cm) were measured from participants in underwear and barefoot. Body mass index (BMI) was calculated as weight (kg) divided by the squared height (m2). |
|  | **Waist Circumference** | Continuous | Waist circumference (cm) was measured from the point midway between the costal margin and the iliac crest and hip circumference at the widest point around the greater trochanter. Measurements were recorded to one decimal place. |
|  |  |  |  |
| Laboratory Analysis | **Glucose** | Continuous | Subjects were asked to fast overnight, and blood samples were taken from the subjects between 8.00 and 11.00 a.m. All samples were analysed at Oulu University Hospital laboratory. Analyses of plasma glucose, serum total cholesterol, HDL-cholesterols and triglycerides were conducted within 24 h, using a Hitachi 911 Chemistry Analyser (Roche, Boehringer Mannheim, Germany ) and analyses of plasma glucose were analysed using a Granutest 250 (Diagnostica Merck, Darmstadt, Germany) at age 31 years and an Advia 1800 (Siemens Healthcare Diagnostics Inc.,Tarrytown, Ny, USA Country) at age 46 years. Samples for assay of serum insulin were stored at −20 °C and analysed within 7 days of sampling using RIA (Pharmacia Diagnostics, Uppsala, Sweden). |
|  | **High Density Lipoproteins Cholesterol (HDL-C)** | Continuous |  |
|  | **Triglycerides (TRI)** | Continuous |  |
|  | **Insulin** | Continuous |  |
|  |  |  |  |
| 31 year follow-up Questionnaires | **Depression** | Continuous | The Hopkins Symptoms Checklist (HSCL) contains 25 questions relating to depression and anxiety [38]. Symptoms are marked from 1 “not at all” to 4 “very much”. Items 2-3, 5-6, 9, 12-15, 17-19, 21-23 were totalled and divided by the number of items (15) to calculate a mean depression score. |
|  | **Anxiety** | Continuous | Items 1, 4, 7, 8, 10-11, 16, 20, 24-25 from the HSCL were totalled and divided by the number of items (10) to calculate a mean anxiety score. |
|  | **Sleep** | Ordinal  (3 response categories) | The HSCL asked participants to rate their difficulty falling asleep. Four response categories ranged from 1 “no difficulties” to 4 “very much difficulty”. Categories 3 and 4 were later combined for this analysis. |
|  | **Functioning** | Continuous | A weighted score was derived from the 15D functioning questionnaire [39]. |
|  | **Optimism** | Continuous | This score was derived from a series of 6 questions. The participant was asked to mark how well each statement applied to them on a scale of perfectly well, well, somewhat, slightly and not at all. Questions were “At uncertain times, I always expect the best outcome”, “If something can go wrong, it will go wrong for me”, “I always have a positive and optimistic attitude”, “I hardly ever expect things to turn out as I would like”, “I hardly ever expect that something good would happen to me” and “I believe that more good things than bad things generally happen to me”. |
|  | **Life Satisfaction** | Ordinal  (5 response categories) | Participants were asked how they felt about their current life situation in general:  1. Very satisfied  2. Quite satisfied  3. Quite unsatisfied  4. Very unsatisfied  5. Cannot say |
|  | **Social Anhedonia** | Continuous | This score was derived from a series of standard questions and scoring system [24,26]. |
|  | **Coping (Active, Adaptive and Passive)** | Continuous | This question was grouped into 4 separate variables based on [40]. Participants were asked to remember a stressful situation in the last month and then asked to respond how they acted to a series of questions. Responses were marked as not at all, to a small extent, to a moderate extent and to a great extent. Active coping was generated using the total score from 5 questions: I focussed on how to proceed, I made an action plan and followed it, I acted in a way that would turn things around for the better, I kept my feet on the ground and persistently pursued my goal and I thought of different solutions to the problem.  Adaptive coping was generated as the total score from 6 questions: I tried to look on the bright side of things, I was shown support and understanding and I accepted it, I tried to identify my role in the emergence of the problem, I tried to find new faith in things, I pondered what is truly important in life and I compared it to my previous experiences.  Passive Coping was generated as the total score of the remaining 4 questions: I tried to make myself feel better by eating, drinking, taking pills etc, I did not let it bother me, I refused to think about it and I hoped it would somehow go away, that time would take care of it for me. |
|  | **Education** | Ordinal  (2 & 3 response categories) | Educational level was derived from a self-reported questionnaire from two separate questions on basic and vocational education and was classified as:  Basic Education  1. Basic school combined two responses 1) less than 9 years of basic school and 2) basic school. This covers ages 7 to 16 years.  2. Matriculation Examination (completed during final year of school, usually aged 18 years)  Further Education  1. University education (also including polytechnic education and unfinished studies)  2. Vocational Training includes vocational training course, vocational school and post-secondary school  3. No Additional Education beyond basic school |
|  | **Employment Status** | Ordinal  (3 response categories) | Participants were asked “Which of the following alternatives best describes your working history?”  Seven response categories were available and for data analysis these were combined into three:  1. Employed continuously, mainly long-term employments and temporary unemployments  2. Both long-term and short-term employments, mainly short-term employments, but more employment than unemployment  3. Mainly short-term employments, and more unemployment than employment, most of the employments have been arranged by the governmental support system, I have never been in gainful employment |
|  | **Marital Status** | Binary | Five response categories included married, cohabiting, single, legal separation or divorce, widow. These were re-categorised for the data analysis into:  1. Married, cohabiting  2. Single, legal separation or divorce, widowed |
|  | **Occupation** | Categorical (3 response categories) | Participants were asked to write their present occupation and responses were coded. Nine response categories were available and for data analysis these were combined into five:  1. Professional – entrepreneur, upper and lower white collar  2. Manual worker/Farmer – blue collar  3. Not in labour force – students, pensioners  4. Unemployed – or others |
|  | **Home Ownership** | Binary | Seven response categories were combined into two for analysis:  1. Own home  2. Do not own home |
|  | **Household Income** | Continuous | Original household income was recorded in Finnish Marks as was the currency in 1997, however a conversion has been applied to convert this figure to Euros (5.679). OECD square root scale was then applied to modify this figure accounting for the number of adults and children in the household. (41) Modified Income = Household Income / (√number of people in household) |
