# Supplementary material for: Understanding the complexity of glycaemic health: systematic bio-psychosocial modelling of fasting glucose in middle-age adults; a DynaHEALTH study
Source: Int J Obes (Lond). 2018 Aug 17;43(6):1181–92. doi: 10.1038/s41366-018-0175-1 (PMC6760581; doi:10.1038/s41366-018-0175-1)
Supplement: Supplementary file 6 — Supplementary Table 5 [file 41366_2018_175_MOESM6_ESM.docx]

**S5: Exploratory Factor Analysis for all bio-psychosocial variables at 31 years which were significantly associated with fasting glucose at 46 years (n=5078).**

|  | **3 Factors** | | | **4 Factors** | | | | **5 Factors** | | | | |
| --- | --- | --- | --- | --- | --- | --- | --- | --- | --- | --- | --- | --- |
| **Model Fit** | **χ2 = 2228.67, df = 117, p<0.001;**  **CFI = 0.91; TLI = 0.87; RMSEA = 0.084** | | | **χ2 = 1530.82, df = 101, p<0.001;**  **CFI = 0.94; TLI = 0.90;**  **RMSEA = 0.074** | | | | **χ2 = 934.98, df = 86, p<0.001;**  **CFI = 0.96; TLI = 0.91;**  **RMSEA = 0.062** | | | | |
| **Variables** | **1** | **2** | **3** | **1** | **2** | **3** | **4** | **1** | **2** | **3** | **4** | **5** |
| Insulin | 0.115 | **0.618** |  | 0.179 | **0.646** | -0.029 | -0.081 | 0.052 | **0.479** | **-0.447** |  | 0.247 |
| Waist circumference |  | **0.773** |  |  | **0.689** | 0.173 |  | 0.025 | **0.849** | 0.015 | 0.081 | -0.074 |
| BMI | -0.063 | **0.752** | 0.083 | -0.049 | **0.712** | 0.072 | 0.059 | -0.069 | **0.919** | 0.050 | -0.029 | -0.017 |
| SBP | 0.191 | **0.418** | -0.158 |  | 0.025 | **0.838** | -0.067 | 0.033 | 0.033 |  | **0.814** | -0.071 |
| DBP | 0.102 | **0.414** | -0.072 | -0.080 | 0.130 | **0.655** | 0.021 | -0.082 | 0.112 |  | **0.678** | 0.037 |
| HDL-C | -0.055 | **-0.420** |  | -0.067 | **-0.399** | -0.039 |  | -0.098 | **-0.387** | 0.072 |  |  |
| TG | -0.029 | **0.563** | 0.099 |  | **0.584** | -0.046 | 0.054 |  | **0.437** | -0.250 |  | 0.202 |
| Glucose | 0.274 | 0.279 | -0.115 | 0.218 | 0.218 | 0.112 | -0.105 | 0.215 | 0.118 | -0.223 | 0.128 | 0.028 |
| Basic education | **0.694** | 0.114 | -0.007 | **0.697** | 0.143 |  | -0.027 | **0.778** |  | -0.097 |  | -0.030 |
| Higher education | **0.452** |  |  | **0.458** | 0.051 |  |  | **0.487** |  |  |  |  |
| Occupation type | **0.605** |  | 0.118 | **0.595** |  | 0.040 | 0.106 | **0.598** |  | 0.146 |  |  |
| Income | **0.472** | -0.099 | 0.246 | **0.468** | -0.049 |  | 0.232 | **0.432** | 0.043 | **0.401** | -0.064 |  |
| Employment status | 0.233 | -0.161 | **0.335** | 0.253 | -0.047 | -0.133 | **0.309** | 0.250 | 0.056 | **0.359** | -0.164 | 0.108 |
| Marital Status | 0.192 |  | **0.364** | 0.067 | -0.151 | **0.329** | **0.418** |  | -0.058 | **0.360** | **0.314** | 0.267 |
| Home Ownership | 0.119 |  | **0.334** | 0.043 | -0.093 | 0.163 | **0.357** |  |  | **0.368** | 0.137 | 0.205 |
| Depression |  | -0.054 | **0.704** | -0.102 | 0.049 | -0.052 | **0.717** | -0.028 |  |  | -0.094 | **0.740** |
| Sleep Quality | -0.068 |  | **0.575** | -0.153 | 0.091 | -0.037 | **0.585** | -0.071 |  | -0.049 | -0.044 | **0.607** |
| Life Satisfaction | 0.199 |  | **0.580** | 0.073 |  | 0.175 | **0.622** | 0.114 |  | 0.202 | 0.146 | **0.522** |
| Adaptive Coping | -0.094 | -0.096 |  | -0.066 |  | -0.093 |  | -0.089 |  |  | -0.096 |  |

Model fit statistics and geomin factor loadings for 3-5 factor structures. Results shown are for the full dataset. Empty squares represent insignificant loadings. All shaded squares represent loadings greater than 0.3. Model fit statistics: χ2, Chi-Square; df, degrees of freedom; CFI, comparative fit index; TLI, Tucker-Lewis index; RMSEA, root mean square error of approximation. BMI, body mass index; HDL-C, high density lipoprotein cholesterols; TG, triglycerides; SBP, systolic blood pressure; DBP, diastolic blood pressure.
