# Supplementary material for: Understanding the complexity of glycaemic health: systematic bio-psychosocial modelling of fasting glucose in middle-age adults; a DynaHEALTH study
Source: Int J Obes (Lond). 2018 Aug 17;43(6):1181–92. doi: 10.1038/s41366-018-0175-1 (PMC6760581; doi:10.1038/s41366-018-0175-1)
Supplement: Supplementary file 9 — Supplementary Table 8 [file 41366_2018_175_MOESM9_ESM.docx]

**S8: Association of 31-year factor scores with F-Glucose at 46 years (Model 1: unadjusted for sex; Model 2: adjusted for sex).**

|  | **Model 1** | | | | | **Model 2** | | | | |
| --- | --- | --- | --- | --- | --- | --- | --- | --- | --- | --- |
|  | **Estimate (Beta, 95% CI)** | **P value** | **R^2^** | **AIC** | **BIC** | **Estimate (Beta, 95% CI)** | **P value** | **R^2^** | **AIC** | **BIC** |
| **Socioeconomic** | 0.178 (0.136, 0.219) | **<0.001** | 0.014 | 11946.4 | 11966.0 | 0.116 (0.075, 0.158) | **<0.001** | 0.067 | 11666.3 | 11692.4 |
| Socioeconomic^M^ | 0.060 (0.018, 0.102) | **0.005** | 0.077 | 11610.9 | 11637.1 | 0.035 (-0.007, 0.077) | 0.100 | 0.106 | 11451.0 | 11483.7 |
| Socioeconomic^P^ | 0.196 (0.151, 0.241) | **<0.001** | 0.014 | 11944.0 | 11970.2 | 0.113 (0.068, 0.158) | **<0.001** | 0.067 | 11668.2 | 11700.8 |
| Socioeconomic^BP^ | 0.130 (0.089, 0.172) | **<0.001** | 0.045 | 11785.4 | 11811.5 | 0.094 (0.053, 0.136) | **<0.001** | 0.079 | 11599.4 | 11632.1 |
| Socioeconomic^M+P^ | 0.050 (0.003, 0.096) | **0.036** | 0.077 | 11611.8 | 11644.4 | 0.011 (-0.035, 0.057) | 0.649 | 0.107 | 11447.0 | 11486.2 |
| Socioeconomic^M+BP^ | 0.060 (0.018, 0.102) | **0.005** | 0.079 | 11604.2 | 11636.9 | 0.035 (-0.007, 0.077) | 0.098 | 0.106 | 11452.7 | 11491.9 |
| Socioeconomic^P+BP^ | 0.131 (0.086, 0.177) | **<0.001** | 0.045 | 11787.3 | 11820.0 | 0.081 (0.036, 0,127) | **<0.001** | 0.080 | 11599.6 | 11638.8 |
| Socioeconomic^M+P+BP^ | 0.047 (0.001, 0.094) | **0.045** | 0.079 | 11604.6 | 11643.8 | 0.011 (-0.035, 0.057) | 0.652 | 0.107 | 11448.5 | 11494.2 |
| **Metabolic** | 0.112 (0.101, 0.123) | **<0.001** | 0.076 | 11616.7 | 11636.3 | 0.090 (0.079, 0.101) | **<0.001** | 0.105 | 11451.7 | **11477.9** |
| Metabolic^S^ | 0.107 (0.096, 0.119) | **<0.001** | 0.077 | 11610.9 | 11637.1 | 0.087 (0.076, 0.099) | **<0.001** | 0.106 | 11451.0 | 11483.7 |
| Metabolic^P^ | 0.112 (0.102, 0.123) | **<0.001** | 0.076 | 11614.2 | 11640.3 | 0.090 (0.079, 0.101) | **<0.001** | **0.107** | **11445.2** | 11477.9 |
| Metabolic^BP^ | 0.100 (0.087, 0.113) | **<0.001** | 0.077 | 11785.4 | 11811.5 | 0.088 (0.075, 0.101) | **<0.001** | 0.105 | 11599.4 | 11632.1 |
| Metabolic^S+P^ | 0.108 (0.097, 0.120) | **<0.001** | 0.077 | 11611.8 | 11644.4 | 0.089 (0.078, 0.101) | **<0.001** | **0.107** | 11447.0 | 11486.2 |
| Metabolic^S+BP^ | 0.095 (0.082, 0.109) | **<0.001** | 0.079 | 11604.2 | 11636.9 | 0.085 (0.072, 0.099) | **<0.001** | 0.106 | 11452.7 | 11491.9 |
| Metabolic^P+BP^ | 0.100 (0.087, 0.113) | **<0.001** | 0.078 | 11606.6 | 11639.2 | 0.088 (0.074, 0.101) | **<0.001** | **0.107** | 11446.7 | 11485.9 |
| Metabolic^S+P+BP^ | 0.096 (0.083, 0.110) | **<0.001** | 0.079 | 11604.6 | 11643.8 | 0.087 (0.073, 0.100) | **<0.001** | **0.107** | 11448.5 | 11494.2 |
| **Psychosocial** | 0.042 (-0.022, 0.019) | 0.201 | <0.001 | 12013.8 | 12033.4 | 0.081 (0.019, 0.144) | **0.011** | 0.062 | 11690.3 | 11716.5 |
| Psychosocial^S^ | -0.074 (-0.143, -0.004) | **0.038** | 0.014 | 11944.0 | 11970.2 | 0.012 (-0.056, 0.081) | 0.724 | 0.067 | 11668.2 | 11700.8 |
| Psychosocial^M^ | 0.067 (0.005, 0.129) | **0.034** | 0.076 | 11614.2 | 11640.3 | 0.091 (0.030, 0.152) | **<0.001** | 0.107 | 11445.2 | 11477.9 |
| Psychosocial^BP^ | 0.077 (0.014, 0.141) | **0.017** | 0.039 | 11817.1 | 11843.2 | 0.098 (0.036, 0.160) | **0.002** | 0.077 | 11609.9 | 11642.6 |
| Psychosocial^S+M^ | 0.037 (-0.031, 0.105) | 0.287 | 0.077 | 11611.8 | 11644.4 | 0.085 (0.017, 0.152) | **0.014** | 0.107 | 11447.0 | 11486.2 |
| Psychosocial^S+BP^ | -0.004 (-0.073, 0.066) | 0.921 | 0.045 | 11787.3 | 11820.0 | 0.047 (-0.021, 0.116) | 0.175 | 0.080 | 11599.6 | 11638.8 |
| Psychosocial^M+BP^ | 0.074 (0.012, 0.136) | **0.020** | 0.078 | 11606.6 | 11639.2 | 0.092 (0.031, 0.154) | **0.003** | 0.107 | 11446.7 | 11485.9 |
| Psychosocial^S+M+BP^ | 0.045 (-0.024, 0.113) | 0.200 | 0.079 | 11604.6 | 11643.8 | 0.086 (0.018, 0.153) | **0.013** | 0.107 | 11448.5 | 11494.2 |
| **Blood Pressure** | 0.018 (0.016, 0.021) | **<0.001** | 0.038 | 11820.7 | 11840.3 | 0.012 (0.009, 0.015) | **<0.001** | 0.076 | 11617.4 | 11643.5 |
| Blood Pressure^S^ | 0.017 (0.014, 0.019) | **<0.001** | 0.045 | 11785.4 | 11811.5 | 0.011 (0.009, 0.014) | **<0.001** | 0.079 | 11599.4 | 11632.1 |
| Blood Pressure^M^ | 0.005 (0.002, 0.008) | **0.003** | 0.077 | 11785.4 | 11811.5 | 0.001 (-0.002, 0.004) | 0.585 | 0.105 | 11599.4 | 11632.1 |
| Blood Pressure^P^ | 0.018 (0.016, 0.021) | **<0.001** | 0.039 | 11817.1 | 11843.2 | 0.012 (0.010, 0.015) | **<0.001** | 0.077 | 11609.9 | 11642.6 |
| Blood Pressure^S+M^ | 0.005 (0.002, 0.008) | **0.003** | 0.079 | 11604.2 | 11636.9 | 0.001 (-0.002, 0.004) | 0.566 | 0.106 | 11452.7 | 11491.9 |
| Blood Pressure^S+P^ | 0.017 (0.014, 0.019) | **<0.001** | 0.045 | 11787.3 | 11820.0 | 0.011 (0.009, 0.014) | **<0.001** | 0.080 | 11599.6 | 11638.8 |
| Blood Pressure^M+P^ | 0.005 (0.002, 0.008) | **0.002** | 0.078 | 11606.6 | 11639.2 | 0.001 (-0.002, 0.004) | 0.477 | 0.107 | 11446.7 | 11485.9 |
| Blood Pressure^S+M+P^ | 0.005 (0.002, 0.008) | **0.002** | 0.079 | 11604.6 | 11643.8 | 0.001 (-0.002, 0.004) | 0.480 | 0.107 | 11448.5 | 11494.2 |

Model 1: unadjusted; Model 2: adjusted for sex

^S^ adjusted for socioeconomic factor; ^M^ adjusted for metabolic factor; ^P^ adjusted for psychosocial factor; ^BP^ adjusted for blood pressure factor; CI, confidence intervals. Significant P values (P<0.05) are written in bold. Lowest AIC, BIC and highest R^2^ are also highlighted in bold.

Beta = increase in fasting glucose at 46 years caused by one unit increase in factor score.
